# Molecular Level Insight Into the Benefit of Myricetin and Dihydromyricetin Uptake in Patients With Alzheimer’s Diseases

**DOI:** 10.3389/fnagi.2020.601603

**Published:** 2020-10-23

**Authors:** Miaomiao Liu, Hong Guo, Zhongyuan Li, Chenghua Zhang, Xiaoping Zhang, Qinghua Cui, Jingzhen Tian

**Affiliations:** ^1^College of Pharmacy, Shandong University of Traditional Chinese Medicine, Jinan, China; ^2^Affiliated Hospital of Integrated Traditional Chinese and Western Medicine, Nanjing University of Chinese Medicine, Nanjing, China; ^3^Qingdao Academy of Chinese Medicinal Sciences, Shandong University of Traditional Chinese Medicine, Qingdao, China

**Keywords:** Alzheimer’s disease, natural product, Myricetin, Dihydromyricetin, mechanism of action

## Abstract

Alzheimer’s disease (AD) is a neurodegenerative disease with a high incidence rate and complicated pathogenesis. Currently, all anti-AD drugs treat the symptoms of the disease, and with currently no cure for AD. Flavonoid containing natural products, Myricetin (MYR) and Dihydromyricetin (DMY), are abundant in fruits and vegetables, and have been approved as food supplements in some countries. Interestingly, MYR and DMY have been reported to have anti-AD effects. However, the underlying anti-AD mechanism of action of MYR and DMY is complex with many facets being identified. In this review, we explore the benefit of MYR and DMY in AD patients from a molecular level. Their mechanism of action are discussed from various aspects including amyloid β-protein (Aβ) imbalance, neuroinflammation, dyshomeostasis of metal ions, autophagy disorder, and oxidative stress.

## Introduction

Alzheimer’s disease (AD) is a neurodegenerative disease (Barnett, 2019), which can cause patients to gradually lose their ability to live independently, and change their personality and behavior. Most patients with AD die within 10 years of diagnosis, and those patients who survive past this, suffer from declines in cognition, language including speech, and memory. AD not only threatens the patients’ lives and health but also causes serious societal problems (Guo et al., 2019), especially within countries with a growing aging population. Unfortunately, the incidence of AD has dramatically increased in the last few decades. According to the latest report from the Alzheimer’s Association, the number of AD patients in the United States reached 5.8 million in 2020 (Association, 2020). At the same time, they predicted that this number would rise to 13.8 million by mid-century (Association, 2020).

The etiology of AD is complicated and remains unclear, with many factors being reported to be involved in the pathogenesis of AD. The excessive amounts of Aβ and the abnormally phosphorylated tau peptide (Goedert and Spillantini, 2006) are the most studied pathogenesis of AD. The neurotransmitter imbalance in the central nervous system (Guo et al., 2019) such as acetylcholine (ACh) deficiency, the dyshomeostasis of metal ions (Ayton et al., 2015) and the overexpression of MicroRNA (Sarkar et al., 2019) are also thought to be closely related to the development of AD. Furthermore, physiological function disorders, such as oxidative stress, inflammation (Meraz-Ríos et al., 2013), abnormal autophagy and the damage of insulin signaling pathway (Zhang and Hölscher, 2020) were also found to cause serious damage to the brain and can lead to the manifestation of AD (Butterfield and Halliwell, 2019). Other factors are also reported as a driving force in the genesis and development of AD, such as the long-term infections of bacteria and viruses (Bearer and Wu, 2019; Dominy et al., 2019), and the dyshomeostasis of intestinal flora (Friedland, 2015).

Currently, clinically used AD drugs treat the symptoms of AD in affected patients. Donepezil, Rivastigmine and Galantamine are AChE inhibitors while Memantine is an NMDA (N-methyl-D-aspartic acid) receptor antagonist. Notably, GV-971, an oligosaccharide derived from brown seaweed, was approved for use in China at the end of 2019. GV-971 is said to treat AD with a brain-gut axis as the target (Wang X. et al., 2019). In addition, given the crucial role of Aβ in the pathological development of AD, many drugs were developed to control the abnormal accumulation of Aβ. Verubecestat and Tarenflurbil inhibit enzymes within the Aβ biosynthetic pathway, Tramiprosate and Azeliragon inhibit abnormal aggregation of Aβ, monoclonal antibodies (such as Gantenerumab and Aducanumab) and vaccines (such as ACC-001) against Aβ are also used as immunotherapy (Sevigny et al., 2016; Wu, 2019). Most of these candidates failed in phase III clinical trials, mainly due to lack of efficacy against placebo.

## Myricetin and Dihydromyricetin

**FIGURE 1:**
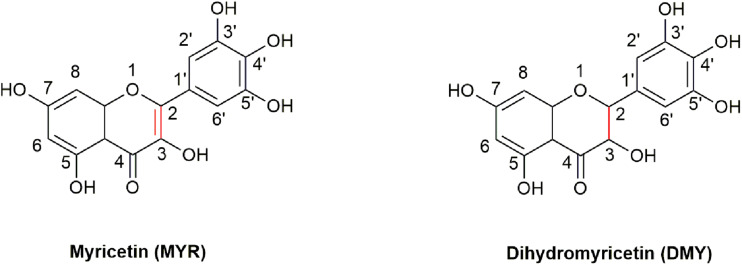
The structures of MYR and DMY. MYR is a flavonol compound, and DMY belongs to dihydroflavonol. DMY is a derivative after introducing “H” to MYR’s 2, 3 binding sites, respectively.

3, 3′, 4′, 5, 5′, 7-Hexahydroxyflavone (Myricetin, MYR, Figure 1) is a flavonoid, which was first isolated from the bark of *Myrica nagi* Thunb. about 200 years ago (Semwal et al., 2016). The appearance of MYR is a light yellow crystal solid. 3, 3′, 4′, 5, 5′, 7-Hexahydroxy-2, 3-dihydroflavanonol (Dihydromyricetin, DMY or DHM) is also known as ampelopsin (AMP) because it was first discovered from *Ampelopsis meliaefolia* (Hand. -Mazz.) W. T. Wang (an Ampelopsis Michx plant) in 1940 (Hou et al., 2015). DMY has a white appearance. MYR is the oxidation product of DMY, thus 2, 3-double bond of DMY is hydrogenated to form MYR (Figure 1).

MYR and DMY are produced in sizable quantities in plants, particularly in some commonly consumed fruits and vegetables. For instance, MYR, has been reported to be abundant in strawberries, apples, spinach, aloe vera, carrots (Zhang et al., 2015), mulberries (Mahmood et al., 2012), etc., and the content of MYR in red wine can reach twice that of resveratrol (Lee et al., 2007; Rodrigo et al., 2011). Dihydromyricetin is widely found in grapes, bayberry, ampelopsis (Clementi et al., 2015), ginkgo and other plants, with the content of DMY in rattan tea particularly is high, often reaching 30–40% (Liu et al., 2019). Moreover, MYR is listed as a health product in Europe, and has been approved by the FDA for pharmaceuticals, foods, and health products in the United States with FYI, a health product containing myricetin being successfully launched (Whitehouse, 2002). Meanwhile, MYR and DMY are the essential ingredients in many health foods or drinks (Semwal et al., 2016; Martínez-Coria et al., 2019) and are known to have an excellent safety profile coupled with the fact that is suitable for human consumption.

Being one of the more well-studied polyphenols, MYR and DMY exhibit a range of interesting biological activities. MYR has been shown to have anti-cancer, anti-oxidant and anti-inflammatory effects (Kang et al., 2011; Zhang et al., 2011). DMY has shown to have better pharmacological effects than MYR, with its anti-temulence (Shen et al., 2012) ability and hepato-protective qualities being well known (Murakami et al., 2004). Interestingly, both MYR and DMY have been found to exhibit anti-AD effects. Moreover, many MYR and DMY containing foods are also reported to have some neuroprotective abilities. Ginkgo and its extracts have long been considered to have a good effect on the treatment of AD (Bader et al., 2018; Li et al., 2018). Aloe vera and mulberry are often considered to have additional anti-dementia effects (Clementi et al., 2015; Liu and Du, 2020). Furthermore, moderate consumption of red wine is also often considered to have anti-aging and antioxidant effects as well as improving blood pressure.

## The Molecular Mechanisms of MYR and DMY in the Alleviation of Hallmarks of Alzheimer’s Disease

Studies have shown that MYR and DMY can significantly improve the learning and memory abilities of animal model of Alzheimer’s disease (Hirohata et al., 2007; Liang et al., 2014). In this section, we explore the underlying molecular mechanisms and potential mode of action of MYR and DMY against the symptoms of AD.

### MYR and DMY Interact With Aβ to Exert Anti-AD Effects

**FIGURE 2:**
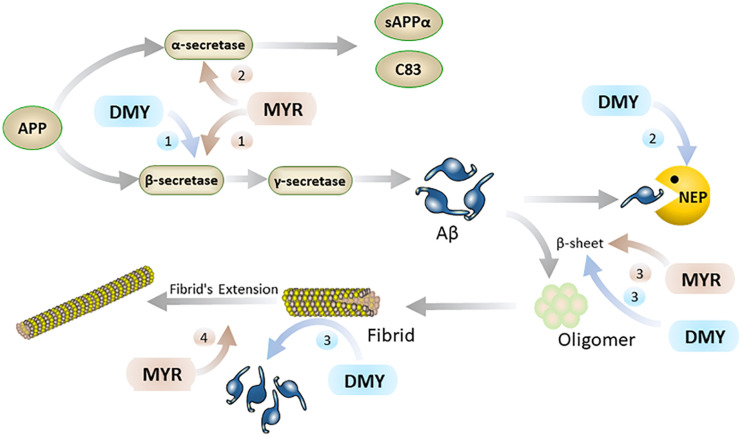
MYR interacts with Aβ through the following 4 pathways (brown): 1. MYR reduces the production of Aβ through inhibition of β-secretase 1; 2. by increasing the level of α-secretase and competitively decomposing APP; 3. inhibiting the β-sheet by binding to Aβ, and preventing the conversion of Aβ monomers into oligomers and fibrils; 4. Inhibiting the extension of the fibrils of Aβ by binding to it. DMY mainly suppresses Aβ in the brain through the following 3 paths (blue): 1. reduce Aβ production by inhibition β-secretase 1; 2. increasing the level of NEP by promoting the expression of NEP gene; 3. inhibiting β-sheet and disassembling Aβ fibrils by binding to Aβ.

Aβ is a hydrolysate of the amyloid protein precursor (APP) (Thinakaran and Koo, 2008). APP has two main metabolic pathways: one is the continuous hydrolysis by β-secretase (BACE-1) and γ-secretase to produce Aβ. In this process, BACE-1 is a key rate-limiting enzyme (Naushad et al., 2019), which is unsurprisingly a potential target for the treatment of AD. The other is the formation of harmless sAPPα and C83 after hydrolysis by α-secretase. Typically, the formation and hydrolysis of Aβ is in dynamic equilibrium. When this dynamic equilibrium is broken, the excessive production and abnormal deposition of Aβ in the brain has been linked to the initiation and progression of AD. It is reported that MYR inhibits the activity of BACE-1 and hinders the production of Aβ (Shimmyo et al., 2008). Inside the active center of BACE-1, the hydroxyl group at the C7 position of the A ring in the MYR structure binds to the aspartate dyad (Asp32 and Asp228) through hydrogen bonding (Chakraborty et al., 2011). Thus, MYR inhibits the activity of BACE-1 to digest APP and thus reduces the production of Aβ. Additionally, MYR has been shown to have increased the level of α-secretase (Shimmyo et al., 2008) which results in an increase in the levels of APP broken down to harmless APP fragments. This causes an overall decrease in the levels of APP that can be used to produce Aβ, thereby indirectly reducing Aβ production. Furthermore, studies also showed that DMY can increase the expression of neprilysin (NEP) (Feng et al., 2018). NEP is a M13 zinc metalloproteinase family protein that can cleave Aβ peptide bonds to decompose Aβ (Kanemitsu et al., 2003; Hersh and Rodgers, 2008). The upregulation of NEP by DMY accelerates the decomposition of Aβ and results in the improvement of AD symptoms (Figure 2).

Secondly, Aβ monomer has a neurotrophic effect, but the oligomers and fibrils of Aβ have severe neurotoxicity (mainly including inflammation, oxidative stress, and destruction of cell membranes) (Jang et al., 2007; Umeda et al., 2011; Zhai et al., 2012). The oligomers and fibrils of Aβ are generated by excess Aβ through β-sheet (Jang et al., 2007) and MYR and DMY can inhibit the formation of this β-sheet (Shimmyo et al., 2008; Jia et al., 2019). The hydroxyl group of MYR forms a hydrogen bond with a carbonyl group and amino group in Aβ (Andarzi Gargari et al., 2018). Secondary structure analysis showed that this interaction between MYR and Aβ could inhibit the β-sheet formation of Aβ, which can prevent Aβ undergoing toxic changes (Andarzi Gargari et al., 2018). Moreover, MYR can also bind with two further sites in Aβ fibrils and inhibit the extension of Aβ (Hirohata et al., 2007; Andarzi Gargari et al., 2018). DMY can combine with the three sites on the Aβ structure to block its molecular conformation and break its intramolecular hydrogen bonds. This not only blocks the β-sheet but also has a dismantling effect on the already formed Aβ fibrils (Jia et al., 2019). Thus, MYR and DMY can hinder the formation of Aβ oligomers, which will reduce the neurotoxicity of Aβ oligomers, and release the symptoms of AD (Figure 2).

### MYR and DMY Show Anti-AD Effects Through Anti-inflammatory

**FIGURE 3:**
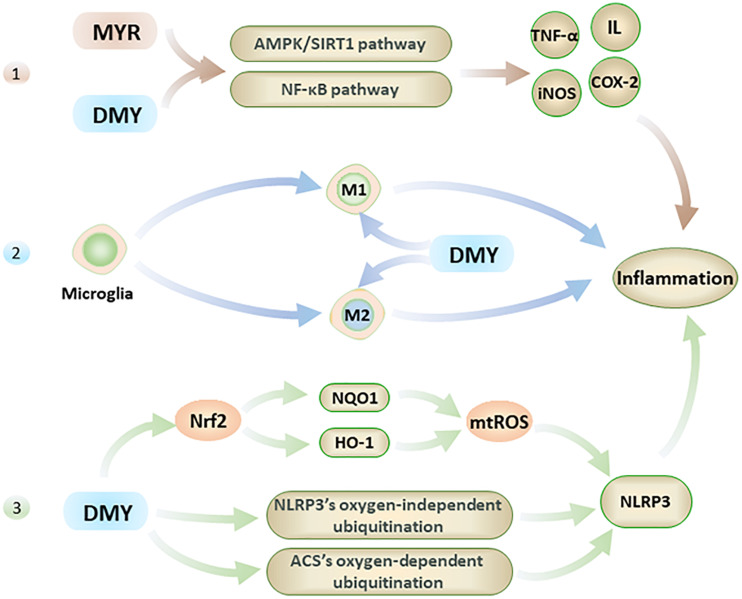
The anti-inflammatory effects of MYR and DMY occur through the following 3 pathways. 1. Through the NF-κB and AMPK/SIRT1 pathway 2. Regulation of microglia, though reduction in M1 levels and increasing M2 level. 3. Activation of Nrf2 to reduce mtROS, and interfere with the ubiquitination process to suppress NLRP3 inflammasome.

Recent research has shown that inflammation is also one of the main causes of AD, although neuroinflammation is usually treated as the result of AD’s pathogenesis (Meraz-Ríos et al., 2013). Interestingly, MYR and DMY can directly reduce the levels of inflammatory factors, inhibit microglia activation, and inhibit NLRP3 (nucleotide-binding oligomerization domain-like receptor protein 3) inflammasome (Figure 3). Eventually, these anti-inflammatory effects of MYR and DMY can help to reduce the symptoms of AD.

Firstly, MYR and DMY can effectively reduce the levels of inflammatory factors such as IL, TNF-α, NF-κB, etc (Jing and Li, 2019). Taking IL-1 as an example, it can not only damage nerve cells but also increase the level of APP to increase the production and accumulation of Aβ (Shadfar et al., 2015). IL-1 can also accelerate the phosphorylation of tau peptide in the brain and further form neurofibrillary tangles (NFTs), and NFTs are another major pathological marker of AD besides Aβ(Shadfar et al., 2015). In addition, IL-1 can also increase the level of other inflammatory factors (such as TNF-α) to further aggravate the inflammatory response and cell damage caused by it (Shadfar et al., 2015). The anti-inflammatory effects of MYR and DMY are mainly to reduce the levels of IL, TNF-α, iNOS, COX-2, and other inflammatory factors in the brain by interfering with the NF-κB signaling pathway and alleviate the damage of these inflammatory factors to the nervous system (Jing and Li, 2019). It is also reported that DMY can inhibit neuronal inflammation in AD rats by activating the AMPK/SIRT1 pathway (Sun et al., 2019). It is through interfering with this signaling pathway, that DMY can exert its anti-inflammatory ability while inhibiting the apoptosis of hippocampal nerve cells. This is key to treating AD, as the hippocampus of the brain regarded as the main area which of the brain that controls memory.

Secondly, microglia are the smallest glial cells in the central nervous system. Under normal conditions, microglial cells have beneficial nutritional and immunological effects, but activated microglial cells play an essential role in the development of neuroinflammation in AD patients’ brains (Browne et al., 2013). Activated microglial cells have two types: M1 and M2. M1 can promote the development of inflammation, while M2 can inhibit the development of inflammation (Sondag et al., 2009). For microglia, DMY not only inhibits its activation (Jang et al., 2007) but also has an excellent inhibitory effect on the neuroinflammation caused by activated microglia (Weng et al., 2017). Specifically, DMY can inhibit the transformation of microglia to M1 and promote its transformation to M2 (Jang et al., 2007). By reducing the level of M1, the inflammatory response is reduced; meanwhile, increasing the level of M2 can further reduce the damage caused by neuroinflammation by exerting its anti-inflammatory ability.

Lastly, in the pathological process of AD, NLRP3 in the central nervous system can be activated by Aβ and aggravate symptoms of AD (Heneka et al., 2013). Studies have confirmed that Aβ levels can be effectively reduced by inhibiting NLRP3 or knocking out related genes, and the impairment of spatial memory ability of mice with Alzheimer’s disease also can be alleviated in this way (Heneka et al., 2013; Tan et al., 2013). At present, the NLRP3 in the central nervous system is considered an effective target for AD’s treatment (Saresella et al., 2016). DMY can inhibit the expression and activation of NLRP3 (Jang et al., 2007) with DMY increasing the levels of HO-1 and NQO1 by activating Nrf2, which in turn reduces the level of mtROS which leads to the activation of NLRP3 being inhibited (Hu et al., 2018). Furthermore, MYR also can inhibit NLRP3 activation by inhibiting ACS’s oxygen-dependent ubiquitination and promoting oxygen-independent ubiquitination of NLRP3 (Chen et al., 2019). The inhibitory effect of MYR and DMY on the NLRP3 can help reduce neuroinflammatory damage in the brain of AD patients and reduce the level of Aβ to a certain extent.

### MYR and DMY Exert an Anti-AD Effect Through Antioxidant

Oxidative stress reflects an imbalance between the excessive production and incorporation of free radicals and the dynamic ability of a biosystems to detoxify reactive intermediates (Jiang et al., 2016). It is one of the direct causes of aging and a known cause of AD (Jiang et al., 2016). The brain has high oxygen consumption and low antioxidant capacity, which makes it particularly vulnerable to oxidative stress (Guo et al., 2019). Free radicals and ROS are the two main pathways used by oxidative stress to directly damage nerve cells. The free radical contains unpaired electrons, making it in an extremely unstable and highly reactive toward neighboring molecules. This will cause the neighboring molecules to become a new free radical, which in turn reacts with neighboring molecules to produce a free radical chain reaction, causing severe oxidative damage to the brain.

As flavonoids, the pyrogallol structure in the B ring of both MYR and DMY is key to their antioxidant effect (Mendes et al., 2015). They can combine with radicals to form stable semiquinone radicals, thus interrupting the radical chain reaction (Zhang and Chen, 2000). *In vitro* experiments have shown that very low concentrations (0.1, 0.2 μmol/L) of MYR can effectively inhibit the generation of ROS and protect cells from damage caused by oxidative stress (Barzegar, 2016). When the concentration of MYR reaches 5 μg/mL, it can achieve 50% clearance of ROS and 20% clearance of DPPH (1,1-diphenyl-2-picrylhydrazyl) radicals; when the concentration of MYR reaches 10 μg/mL, it restores the levels and activities of antioxidant substances such as SOD, CAT, and GSH-Px in cells (Wang et al., 2010). Additionally, research has shown that in the H_2_O_2_-induced cell injury model, MYR can inhibit DNA and lipid damage caused by oxidative stress while regulating the PI3K/Akt and MAPK signaling pathways. This leads to an increase in the levels of anti-apoptotic factors such as Bcl-2 and reduction of pro-apoptotic factors like Bax, caspase-9, and caspase-3, ultimately leading to inhibition of apoptosis induced by oxidative stress (Wang et al., 2010; Li et al., 2016). DMY also has a very good free radical scavenging ability. The difference from MYR mainly lies in the fact that MYR has a better ability to clear ABTS [2, 2’-azino-bis(3-ethylbenzothiazoline-6-sulfonic acid)] while DMY is better at clearing DPPH (Kang et al., 2010).

**FIGURE 4:**
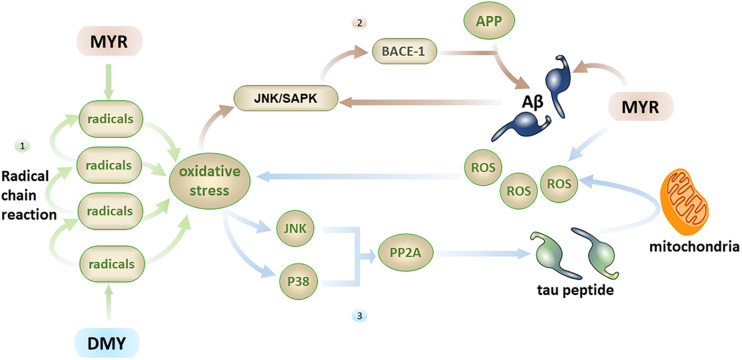
Firstly, the free radical chain reaction can cause severe oxidative stress damage, with MYR and DMY inhibiting this process by scavenging free radicals; secondly, oxidative stress can increase the level of Aβ by activating the JNK/SAPK pathway, and Aβ can further activate this pathway, MYR’s antioxidant and anti-Aβ abilities can disrupt this vicious circle; thirdly, oxidative stress can promote the phosphorylation of tau peptide by inhibiting PP2A, and tau peptide oligomers can increase oxidative stress by destroying mitochondria, the ability of MYR to clear ROS can prevent the increase of oxidative stress.

In addition to direct brain damage, oxidative stress is closely associated with multiple factors related to AD. (1) Aβ has the ability to induce oxidative stress (Butterfield et al., 2010), and oxidative stress can also promote the production of Aβ (Praticò et al., 2001). MYR can inhibit the free radical chain reaction from the source by inhibiting Aβ, thereby reducing the central nervous system damage caused by oxidative stress (Shimmyo et al., 2008). (2) Oxidative stress can activate the JNK/SAPK pathway and subsequently cause elevated BACE-1 levels. Further, BACE-1 will break down the APP into Aβ, which leads to elevated Aβ levels (Tamagno et al., 2005), while an increase in Aβ can further activate the JNK/SAPK pathway (Figure 4) and cause a vicious cycle. Oxidative stress can also promote the phosphorylation of tau peptide by inhibiting PP2A (Praticò et al., 2001; Su et al., 2010; Tang et al., 2017), and oligomers of tau peptide can aggravate oxidative stress by destroying mitochondria (Lasagna-Reeves et al., 2011; Figure 4). MYR and DMY can, therefore, protect neuronal cells through their antioxidant abilities.

### MYR and DMY Play an Anti-AD Role by Regulating Autophagy

**FIGURE 5:**
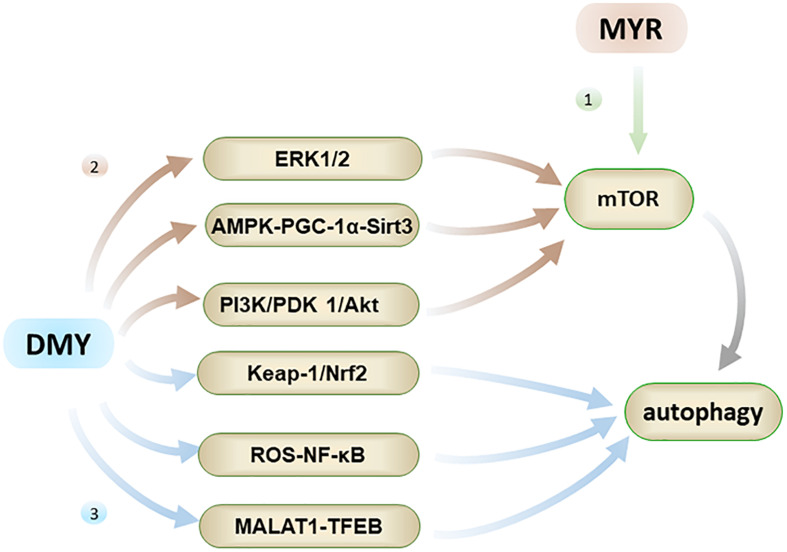
Autophagy has the effect of inhibiting AD. Firstly, MYR can promote autophagy by inhibiting phosphorylation of mTOR; Secondly, DMY can inhibit mTOR through ERK1/2 pathway, AMPK-PGC-1α-Sirt3 pathway, and PI3K/PDK 1/Akt pathway, ultimately promote autophagy; Thirdly, DMY can also promote autophagy through the Keap-1/Nrf2, ROS-NF-κB and MALAT1-TFEB pathways.

Autophagy is the process by which cells eliminate abnormal organelles or denatured protein through lysosomes. For nerve cells, it is difficult to dilute toxins through cell division, so autophagy is a particularly vital process for nerve cells. At the beginning of the autophagy process, the endoplasmic reticulum in the cell sheds part of the biofilm and forms autophagic vesicles (AVs). These AVs encapsulate harmful protein such as Aβ and abnormally phosphorylated tau peptide to form autophagosomes. Subsequently, the autophagosomes are transported along the microtubules by the dynein to the lysosome resulting in harmful protein being degraded by the lysosome (Li et al., 2019; Figure 5). Moreover, recent studies have revealed that the mTOR (mammalian targets of rapamycin) is a crucial signaling factor that regulates cell proliferation, growth, and apoptosis, and it is the core cytokine that regulates cell autophagy.

Both MYR and DMY have an effect on regulating autophagy, which aids in the elimination of abnormal Aβ and tau peptide produced in cells. MYR regulates autophagy mainly through the mTOR pathway by inhibiting mTOR’s phosphorylation (Cao et al., 2018). It has been observed to dose-dependently increase the level of autophagy marker LC3-II and induce formation of autophagosomes (Cao et al., 2018). Through the formation of a large number of autophagosomes, the clearance of Aβ and abnormally phosphorylated tau peptide in the cells is accelerated, thereby alleviating the symptoms of AD (Díaz-Villanueva et al., 2015; Cao et al., 2018). On the other hand, DMY can promote autophagy by inhibiting mTOR, and again this effect is dose-dependent. The regulation of autophagy by DMY is a comprehensive and complicated process, which involves the upstream pathway of mTOR including ERK1/2 (extracellular signal-regulated kinase 1/2), AMPK-PGC-1α-Sirt3 (AMP-activated kinase-peroxisome proliferator-activated receptor coactivator-1α-Sirt3) and PI3K/PDK 1/Akt (class III phosphatidylinositol 3-kinase/phosphoinositide-dependent protein kinase 1/protein kinase B) pathways (Xia et al., 2014; Shi et al., 2015). Furthermore, the regulation of autophagy by DMY also involves the Keap-1/Nrf2, ROS-NF-κB, and MALAT1-TFEB pathways (Qiu et al., 2017; Zhou et al., 2017; Tan et al., 2019). In short, DMY can affect the levels of Aβ and tau peptide in the nervous system through multiple effects on autophagy, and thus has a therapeutic effect on AD.

### MYR and DMY Play an Anti-AD Role by Complexing Metal Ions in the Brain

The imbalance of metal ions in the brain can cause cytotoxicity, oxidative stress damage, abnormal deposition of Aβ, and abnormal phosphorylation of tau peptide, which are closely related to AD. Initially, research conducted regarding AD-related metals generally focused on calcium ions, however, in recent years, an increasing number metal ions (such as copper, iron, and zinc) have been associated with the generation and development of AD (Bush, 2013). Studies have shown that there are many binding sites within Aβ that can bind metal ions (Tian et al., 2018), and there is a notable increase in toxicity of Aβ when this complexation occurs. For example, Zn^2+^ has four binding sites on the structure of Aβ, so even in micromolar concentrations, they can increase the aggregation of Aβ (Boom et al., 2009). Additionally, Zn^2+^ can change Aβ’s structure after binding to Aβ and promote the amyloidosis of Aβ (Guo et al., 2017). Cu^2+^ is also able to bind Aβ and promote its accumulation and precipitation (Cristóvão et al., 2016). This effect is generally related to the relative levels of Cu^2+^/Zn^2+^ and Aβ content (Cristóvão et al., 2016).

**FIGURE 6:**
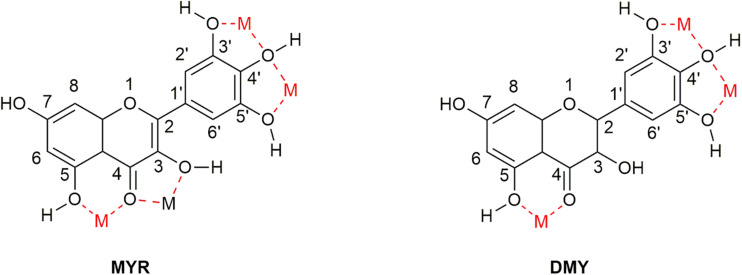
Sites that can complex metal ions in the structure of MYR and DMY. There are four main sites (location of M in the figure) that can be used to chelate metal ions in the structure of MYR since the double bond at the 2–3 position becomes a single bond. DMY is one less than MYR, which can be used to couple metal ions.

As flavonoids, both MYR and DMY have an excellent ability to chelate metal ions (Xu, 2010; Jomová et al., 2019), which can inhibit AD by regulating the concentration of metal ions in the brain. The chelating sites with metal ions of MYR and DMY are shown in Figure 6. It is worth noting that the chelation products have better pharmacological effects (such as antioxidant and anti-inflammatory) than uncomplexed MYR or DMY (Li et al., 2016). MYR has been proven that it could inhibit Aβ aggregation by chelating with Cu^2+^ or Zn^2+^ (DeToma et al., 2011). MYR can regulate the level of metal ions in the brain by complexing with metal ions, and this will reduce the chance of Aβ to combine with metal ions. Moreover, MYR can not only prevent Aβ from binding with metal ions but also disassemble the complexes of Cu^2+^/Zn^2+^-Aβ that have been formed and plunder the metal ions (DeToma et al., 2011). Besides affecting the aggregation of Aβ, Zn^2+^ can also affect the production of Aβ. It can increase the levels of β-secretase and γ-secretase by inhibiting the activity of α-secretase, thereby increasing the level of Aβ in the brain (Garai et al., 2006). This shows that the complexation of MYR and Zn^2+^ can also reduce the level of Aβ by suppressing this phenomenon.

Furthermore, and similar to other divalent ions, Fe^2+^ can bind Aβ and induce the formation of oligomers and fibrils (Boopathi and Kolandaivel, 2016; Lane et al., 2018). Concurrent to this, is the Fenton process (Simunkova et al., 2019), that acts to covert H_2_O_2_ into highly toxic hydroxyl radicals and ROS (Wang T. et al., 2019), and Fe^2+^ serves as a catalyst in this process. As is known these hydroxyl radicals and ROS can cause severe oxidative damage to the central nervous system, and the presence of Fe^2+^ in the central nervous system can dramatically increase this damage. Upon complexation of Fe^2+^ by MYR and DMY, the levels of free Fe^2+^ drops, which results in a reduction in the Fenton reaction. This eventually leads to an overall reduction in oxidative stress damage in the central nervous system. Additionally, Fe^2+^ can also lead to the activation of microglia, which again results in the damage to the nervous system caused by inflammation (Peters et al., 2018). Complexation of Fe^2+^ by MYR and DMY can therefore reduce the risk of this inflammation occurring. Further to this, MYR can also regulate cell to metal ion transport. Wang et al. claimed that MYR could reduce iron levels by inhibiting the expression of transferrin receptor 1 (TrR1) (Wang et al., 2017). This is another way for MYR to adjust the level of metal ions in addition to directly chelating iron ions.

### MYR and DMY Play an Anti-AD Role by Regulating Insulin Signaling Pathways and Intestinal Flora

Insulin signaling pathway damage in the central nervous system can induce AD (Griffin et al., 2005), with AD being called type 3 diabetes. Insulin resistance will cause the levels of β-secretase and γ-secretase to rise, and increase the rate of Aβ generation. Insulin resistance also causes the body to resort to a state of high insulin levels, which in turn causes the body to overexpress insulin degrading enzymes (IDE) which are designed to break down excess insulin. In addition to degrading insulin, IDE also degrades Aβ. Too much insulin will compete with Aβ for the opportunity of being degraded by IDE, resulting in the accumulation of Aβ due to a lack of effective degradation (Dineley et al., 2014). Both MYR and DMY have been shown to effectively inhibit insulin resistance. PTP1β is a protein that negatively regulates the insulin signaling pathway (Lalitha et al., 2020) and can cause insulin resistance. A recent study showed that MYR has a good inhibitory effect on PTP1β(Lalitha et al., 2020). *In vivo* experiments have also shown that the treatment of MYR also increases the body’s sensitivity to insulin by increasing the levels of pIR (phospho-insulin receptor), pIRS1 (phospho-insulin receptor substrate 1) and pAkt (phospho-Akt), thereby increasing the body’s sensitivity to insulin. DMY can treat the dysregulation of insulin signaling pathway by inhibiting the phosphorylation of PPARγ Ser273 and regulating the ERK/CDK5 signaling pathway (Liu, 2017). Further studies have shown that DMY’s ability to regulate intestinal flora also helps to improve insulin resistance (Tong, 2018) with the successful launch of GV971 in China proving the feasibility of treating AD with intestinal flora. Regulation of insulin resistance or other pathological processes in AD patients through intestinal flora may also be potential anti-AD targets of MYR and DMY.

### MYR and DMY Play an Anti-AD Role by Inhibiting AChE

Acetylcholine is a neurotransmitter that plays an important role in the transmission of neural signals and memory formation and the lack of ACh in the central nervous system is a known cause of AD (Guo et al., 2019). AChE has been a major target for drug discovery in recent years with three of the current five AD clinical drugs being AChE inhibitors. Interestingly, both MYR and DMY have been shown to effectively inhibit AChE (Pepeu and Giovannini, 2004; Wang et al., 2017). In the mouse model of Alzheimer’s disease induced by scopolamine, MYR effectively reduced the impairment of learning and memory ability of mice through its AChE inhibitory ability (Kou et al., 2016). The structural characteristics of DMY lead to stronger anti-AChE activity than MYR (Zhao et al., 2012) and exhibits similar inhibitory data when compared to clinical drugs (Tacrine) (Pepeu and Giovannini, 2004), with the added benefit that DMY, as a food materials, has a better safety profile. In addition to AChE, some inflammatory factors also affect the level of ACh, such as IL-1. IL-1 can improve the level of AChE and accelerate the decomposition of ACh (Shadfar et al., 2015), resulting in insufficient ACh content in the brain and affecting memory ability. The anti-inflammatory ability of MYR and DMY can also prevent the loss of ACh.

### MYR and DMY Play an Anti-AD Role by Inhibiting Bacteria and Viruses

Bacteria and viruses also can induce AD, with both *Porphyromonas gingivalis* and *Herpes simplex virus* (HSV) having confirmed links with AD initiation and progression (Bearer and Wu, 2019; Dominy et al., 2019). Gingipains, produced by *Porphyromonas gingivalis*, is a toxic protease related to the phosphorylation of tau peptide and the ubiquitin pathology which can also increase the level of Aβ_42_ in the brain (Bearer and Wu, 2019; Dominy et al., 2019). According to the results reported by Grenier et al., 62.5 to 125 μg/ml of MYR had good inhibitory effect on *Porphyromonas gingivalis*, and this effect may be related to MYR’s ability to chelate iron ions (Grenier et al., 2015). Additionally, MYR can also inhibit the expression of protease and adhesin in *Porphyromonas gingivalis* to reduce the toxic effects of *Porphyromonas gingivalis* (Grenier et al., 2015). Furthermore, MYR also inhibits the inflammatory response caused by *Porphyromonas gingivalis* through activation of NF-κB (Grenier et al., 2015), which can play a role in the adjuvant treatment of AD. Some research data shows that patients with long-term infection with HSV have a higher risk of AD than those patients free of HSV infection. The reason may be that HSV can cause Aβ accumulation and the phosphorylation of tau peptide, which then induces AD. Importantly, HSV was also found in the brain areas seriously affected by AD, which substantiates the above hypothesis (Mangold and Szpara, 2019). The inhibitory effect of MYR on HSV (Lyu et al., 2005) will help patients reduce the risk of AD and help control the status of Aβ and tau peptide.

### Other

MiR (microRNA) is a small-molecule RNA, 21–23 nucleotides in length, which can regulate gene expression. Recent reports showed that overexpression of miR-34a could cause the accumulation of Aβ and the hyperphosphorylation of tau peptide, and eventually lead to AD (Sarkar et al., 2019). Studies have shown that in the brain of Alzheimer’s disease model rats induced by D-gal, DMY can regulate the SIRT1-mTOR signaling pathway by inhibiting miR-34a, and ultimately inhibit D-gal-induced hippocampal neuronal cell damage (Kou et al., 2016).

### Prospects

Alzheimer’s disease is a disease-induced and promoted by a variety of factors. Due to the interaction of multiple reasons, unilateral treatment of AD will be challenging to achieve. As detailed in this review, we know that MYR and DMY can inhibit the excessive production and accumulation of Aβ, inhibit the inflammatory response in the central nervous system, chelate metal ions in the nervous system, regulate autophagy, and inhibit oxidative stress. At the same time, they can also increase ACh levels by inhibiting the activity of AChE, inhibiting the overexpression of miR-34a, reducing the promotion of AD by bacteria and viruses, and alleviating the state of insulin resistance in the central nervous system. They can even regulate the balance of the intestinal flora, which is also a good target for intervention in AD. These combined anti-AD effects also work to improve the symptoms in AD patients. For example, no studies have been shown that MYR and DMY can directly inhibit tau peptide hyperphosphorylation. However, MYR and DMY can also affect tau peptide and NFTs by regulating autophagy, inhibiting oxidative stress, and improving insulin resistance.

Health products and healthy diets are well accepted to regulate human health and prevent diseases. MYR and DMY have better toxicity profiles and exist in a variety of foods. FYI’s approval by the FDA also shows the feasibility of using MYR and DMY for health products. Therefore, we believe that the uptake of MYR and DMY in daily diets to develop health products or foods with the ability to prevent and improve AD has great potential.

## Author Contributions

All the authors listed have made some contributions to the manuscript and approved for publication.

## Conflict of Interest

The authors declare that the research was conducted in the absence of any commercial or financial relationships that could be construed as a potential conflict of interest.
